# An atypical concurrent occurrence of parathyroid adenoma and micropapillary thyroid carcinoma: First case reported in Saudi Arabia

**DOI:** 10.1016/j.ijscr.2023.108199

**Published:** 2023-04-21

**Authors:** Ahmed Hafez Mousa, Moshiur Rahman, Hussain Raeid Alsadeq, Zain Zuhair Albukhari, Abdullatif Sheikh Ibrahim, Islam Khaled

**Affiliations:** a Department of Surgery, Saudi German Hospitals, Jeddah, Saudi Arabia; b Department of Surgery, Faculty of Medicine, Suez Canal University Hospitals, Ismailia, Egypt; c College of Medicine and Surgery, Batterjee Medical College, Jeddah, Saudi Arabia

**Keywords:** Parathyroid adenoma, Papillary thyroid carcinoma, Surgical excision

## Abstract

**Background:**

Papillary thyroid carcinoma (PTC) is the most frequent endocrine cancer and most common thyroid cancer. The concurrent occurrence of both tumors however is a very rare occasional finding. Surgical treatment via excision is the only definitive.

Our study aims to highlight a rare occurrence of concurrent parathyroid adenoma and micropapillary thyroid carcinoma.

**Case presentation:**

We describe a 36-year-old female who presented to the outpatient clinic with a left thyroid nodule. Both a Tc-99m-MIBI parathyroid scan and Tc-99m thyroid scan were performed. A left total thyroidectomy was performed then subsequently the parathyroid adenoma was localized. Intra-operative parathyroid hormone decreased by >50 % from 531.5 pg/ml iPTH Stat to 39.8 pg/ml iPTH Stat which is diagnostic for proper localization.

Two specimens were sent for histopathological evaluation. Histopathological evaluation of the first specimen confirmed the diagnosis of parathyroid adenoma. Histopathological evaluation of the second specimen revealed that the presence of papillary microcarcinoma of a size of 0.8 cm and pathologic staging to be pT1a, pNx, pMx.

**Conclusion:**

To our knowledge, this is the first case of concurrent occurrence of parathyroid adenoma and micropapillary thyroid carcinoma reported in the Kingdom of Saudi Arabia.

Intraoperatively, management was done by via excision and confirmation of the parathyroid localization was done via intraoperative parathyroid hormone level measurement.

We recommend more extensive studies to identify any possible patterns or predictors of finding these two concurrent tumors.

## Highlights

• Papillary thyroid carcinoma (PTC) is the most frequent endocrine cancer and most common thyroid cancer. The concurrent occurrence of both tumors however is a very rare occasional finding
• A clear pathogenetic relationship between PTC and parathyroid adenoma has not been well studies yet
• To our knowledge this is the first case reported in Saudi Arabia

## 1 Introduction

Papillary thyroid carcinoma (PTC) is the most frequent endocrine cancer and most common thyroid cancer [1]. A single parathyroid adenoma is responsible for 80–85 % of hyperparathyroidism [2]. The concurrent occurrence of both tumors however is a very rare occasional finding. Surgical treatment via excision is the only definitive cure [3]. Detection of the tumor could be carried out by various imaging modalities. Ultrasounds may help localization of parathyroid diseases but difficulties in diagnosis show if the parathyroid adenoma is intrathyroidal 99mTc-MIBI scanning has been frequently relied on recently in detection of such cases and has shown to be highly successful [4]. A clear pathogenetic relationship between PTC and parathyroid adenoma has not been well studies yet [5], [6]. Our study aims to highlight a rare occurrence of concurrent parathyroid adenoma and micropapillary thyroid carcinoma. This work has been reported in line with the SCARE 2020 criteria [23] (Table 1).

**Table 1:** Summary of literature review for previously reported cases of co-occurring parathyroid adenoma and micropapillary thyroid carcinoma.

Reference	Country	Sex	Age (years)	Size of the excised tumor (cm)	Thyroidectomy	iPTH (pg/ml)	
						Preoperative	Postoperative
Our patient	Kingdom of Saudi Arabia	Female	36	0.8 cm	Total	531.5	39.8
Calis H et al. [24]	Turkey	Female	42	0.9 cm	Unilateral lobectomy	N\A	N\A
Calis H et al. [24]	Turkey	Female	76	0.8 cm	Unilateral lobectomy	N\A	N\A
Calis H et al. [24]	Turkey	Male	32	1.4 cm	Total	N\A	N\A
Calis H et al. [24]	Turkey	Female	48	2 cm	Total	N\A	N\A
Calis H et al. [24]	Turkey	Female	50	1.5 cm	Total	N\A	N\A
Yang, J. et al. [25]	China	Male	53	1.2	Left lobectomy	220	3.0
Kutlutürk K et al. [26]	Turkey	Female	45	N\A	Total	N\A	N\A
Kutlutürk K et al. [26]	Turkey	Male	39	N\A	Total	N\A	N\A
Kutlutürk K et al. [26]	Turkey	Female	47	N\A	Total	N\A	N\A
Kutlutürk K et al. [26]	Turkey	Female	62	N\A	Total	N\A	N\A
Kutlutürk K et al. [26]	Turkey	Female	60	N\A	Total	N\A	N\A
GÜREL, Bora et al. [27]	Turkey	Female	76	0.8 cm	Left total and right subtotal thyroidectomy	N\A	N\A
Al-Yahri O et al. [28]	Qatar	Female	61	2 cm	Right hemithyroidectomy	111	N\A
Sharfudeen S et al. [29]	Kuwait	Female	45	1 × 1 × 1.3 cm	Left partial thyroidectomy	41	N\A
Mazeh H et al. [30]	United State	Male	49	0.1 cm	Total	1470	Intra operative, 20 min after removal of parathyroid gland 97.7

## 2 Case presentation

**Fig. 1:**
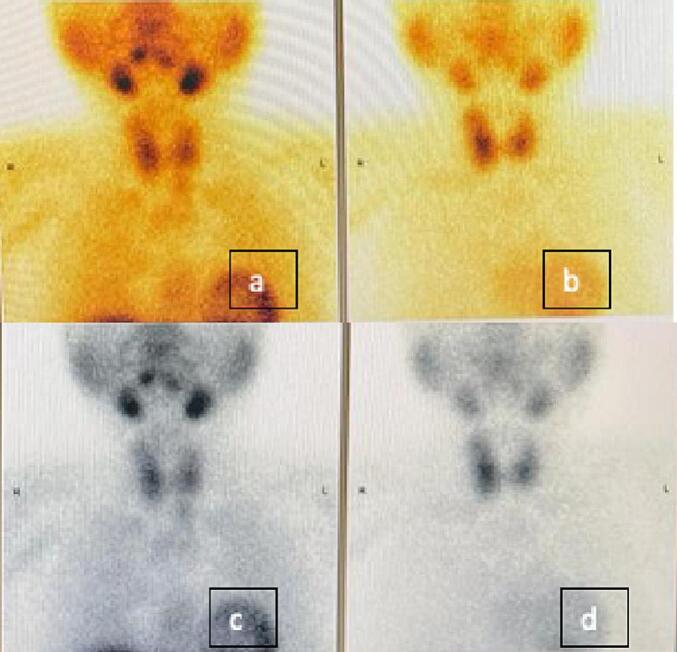
(a and c) Tc-99m-MIBI parathyroid scans (c and d) Tc-99m thyroid scans.

**Fig. 2:**
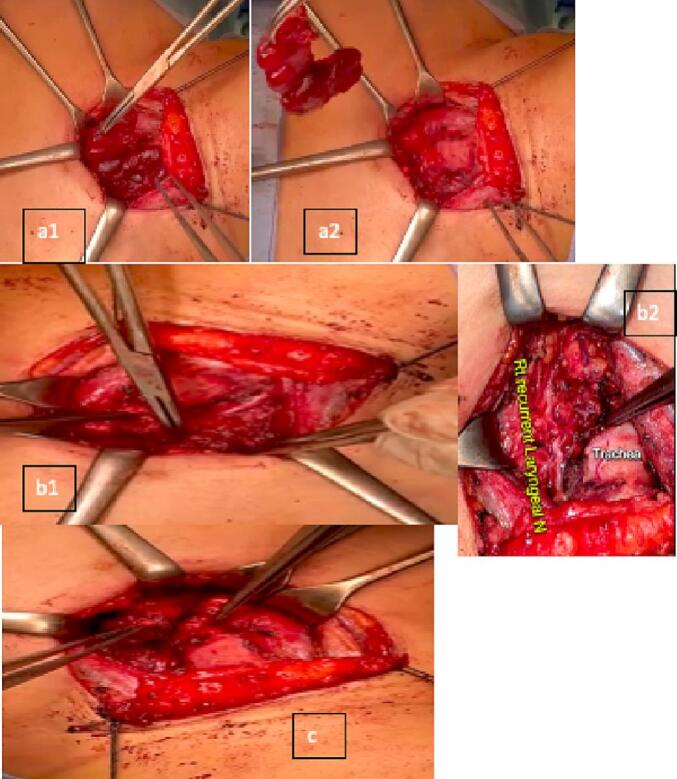
(a) Left total thyroidectomy (a1) before removal of the gland (a2) after removal of the gland (b) the whole course of the recurrent laryngeal nerve (b1) the recurrent laryngeal nerve in relation to the superior mediastinum (b2) recurrent laryngeal nerve in relation to the trachea (c) the parathyroid gland that will be sent for histopathologic evaluation.

**Fig. 3:**
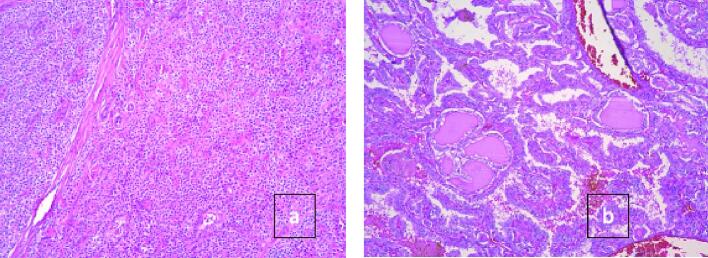
(a) Parathyroid adenoma composed of chief cells (b) thyroid papillary carcinoma.

We describe a 36-year-old female who presented to the outpatient clinic with a left thyroid nodule. Initial labs were requested at the first visit, and they included thyroid function assessment and serum prolactin assessment. Thyroid function was assessed via measurement of the thyroid stimulating hormone (TSH) and was found to be 0.78 uIU/ml which is within the normal reference range (0.35–4.9 uIU/ml). Serum prolactin was found to be elevated with a level of 27.34 ng/ml which is above the reference range (5.18–26.53 ng/ml). The patient had no previous personal history of hyperparathyroidism but no any other co-morbidities and had no family history of any similar presentation. Fine needle aspiration (FNAC) from the prominent left thyroid nodule, grossly three syringes contained 0.1 ml hemorrhagic fluid where seen, and cytology was suspicious for a follicular neoplasm of Hürthle cell type. A preoperative ultrasound of the thyroid was performed and showed a left paratracheal infra-thyroid lesion which raised suspicion of for a left parathyroid adenoma, given the history of hyperparathyroidism associated. Both a Tc-99m-MIBI parathyroid scan (Fig. 1a and c) and Tc-99m thyroid scan (Fig. 1b and d) were performed. A left total thyroidectomy was performed (Fig. 2a) first, then subsequently the parathyroid adenoma was localized. For localization of the parathyroid adenoma, first the recurrent laryngeal nerve was identified and dissected through its course starting from berry's ligament down to the superior mediastinum (Fig. 2b). The parathyroid was deep down in the mediastinum so it was pulled upward and then ligated from its pedicle (Fig. 2c). Intra-operative parathyroid hormone decreased by >50 % from 531.5 pg/ml iPTH Stat to 39.8 pg/ml iPTH Stat which is diagnostic for proper localization. Two specimens were sent for histopathological evaluation. The first specimen was from the left inferior parathyroid excision. Gross evaluation showed fibrofatty tissue and cut sections showed capsulated brownish nodules. Histopathological evaluation confirmed the diagnosis of parathyroid adenoma (Fig. 3a). The second specimen was from the total thyroidectomy. Gross evaluation was done on two separate thyroid lobes. Grossly, both lobes showed ill-defined whitish nodules. Histopathological evaluation revealed that the presence of papillary microcarcinoma of a size of 0.8 cm and pathologic staging to be pT1a, pNx, pMx.

## 3 Discussion

The simultaneous presence of parathyroid adenoma and papillary thyroid carcinoma is an extremely rare occurrence with very few cases documented in the literature. The first documented case of concurrent thyroid and parathyroid pathologies was reported in 1947 [7], [8]. The frequency of isolated parathyroid adenoma relatively common and the rate at which parathyroidectomy is successful for such cases is >95 % [9]. On the other end, PTC is the most common histological type of differentiated malignancy of the thyroid [9]. However, the occurrence of both pathologies together is a very rare entity. Diagnosis of the concurrence could be a challenging task. In our patient utilization of Tc-99m-MIBI scan for the parathyroid gland and Tc-99m for the thyroid gland was done. Two cases reported by Domenico Rubello et al. [10] which had the same co-existence also utilized the same imaging modalities for detection of the pathologies. The double phase imaging procedure with Tc-99m-MIBI was developed by Taillefer at al [11]. Cinamon, U., et al. compared the incidence of thyroid carcinoma among different groups with various stages of hyperparathyroidism and they concluded that detection of thyroid carcinoma at the time of parathyroidectomy is related to risk factors not associated with hyperparathyroidism [12]. Parathyroidectomy is the definitive treatment approach, however success rates depend on the accurate preoperative localizations [13]. In our patient accurate localization was confirmed intraoperatively by observation of >50 % drop in the iPTH Stat level which is diagnostic for proper localization. Ultrasonography has been suggested as step in the imaging done to patients with such presentations. It is commonly done following a presentation of a neck swelling. However, it has shown to be relatively insensitive [14]. In our patient, no significant findings as well were detected by ultrasound further supporting the lack of sensitivity provided by this imaging modality. Some studies however reported that a combination of SPECT/CT and ultrasonography can accurately localize intrathyroidal parathyroid adenomas [15], [16], [17], [18], [19], [20], [21], [22].

## 4 Conclusion

To our knowledge, this is the first case of concurrent occurrence of parathyroid adenoma and micropapillary thyroid carcinoma reported in the Kingdom of Saudi Arabia. Although precise localization and management of such conditions may be challenging, in our patient utilization of Tc-99m-MIBI for the parathyroid and Tc-99m for the thyroid was shown to be efficient preoperatively. Intraoperatively, management was done by via excision and confirmation of the parathyroid localization was done via intraoperative parathyroid hormone level measurement. We recommend that more awareness towards the possibility of concurrent occurrence of these two tumors to be investigated. Additionally, we recommend more extensive studies to identify any possible patterns or predictors of finding these two concurrent tumors.

## Consent

Written informed consent was obtained from the patient for publication of this case report and accompanying images. A copy of the written consent is available for review by the Editor-in-Chief of this journal on request.

## Provenance and peer review

Not commissioned, externally peer-reviewed.

## Ethical approval

Ethical approval is not applicable.

## Funding

No funding received.

## Guarantor

Ahmed Hafez Mousa.

## Research registration number

1. Name of the registry: N/A
2. Unique identifying number or registration ID: N/A
3. Hyperlink to your specific registration: N/A.

## CRediT authorship contribution statement

Ahmed Hafez Mousa: Study concept, design, data collection, data analysis, data interpretation, and paper writing
Islam Khaled: Operating attending surgeon
Moshiur Rahman, Hussain Raeid Alsadeq, Zain Zuhair Albukhari, Abdullatif Sheikh Ibrahim: Sharing in part of the paper writing.

## Conflicts of interest

Not applicable.
